# Towards Identifying Objectivity in Short Informal Text

**DOI:** 10.3390/e27060583

**Published:** 2025-05-30

**Authors:** Chaowei Zhang, Cheng Zhao, Zewei Zhang, Yuchao Huang

**Affiliations:** 1 The Department of Information Engineering, Yangzhou University, Yangzhou 225012, China; mz120230977@stu.yzu.edu.cn; 2 The Department of Computer Science and Software Engineering, Auburn University, Auburn, AL 36849, USA; zzw475622@gmail.com; 3 The Future Design Laboratory of Zhejiang University, Hangzhou 310027, China; huangyuchao@zju.edu.cn

**Keywords:** short informal texts, objectivity learning, relational triples, large language models

## Abstract

Short informal texts are increasingly prevalent in modern communication, often containing fragmented grammar, personal opinions, and limited context. Traditional NLP tasks for the texts ordinarily focus on the subjective aspect learning, such as sentiment analysis and polarity classification. The study of learning objectivity from the texts is similarly significant, which can benefit many real-world applications including information filtering, content verification, etc. Unfortunately, this study is not being explored. This paper proposes a novel task that aims at identifying objectivity in short informal texts. Inspired by the characteristics of objective statements that normally need complete syntax structures for knowledge expression and delivery, we try to leverage the viewpoint of subjects (U), the tense of predicates (V), and the viewpoint of objects (O) as critical factors for objectivity learning. Upon that, we further propose a two-stage objectivity identification approach: (1) a UVO quantification module is implemented via a proposed OpenIE and large language model (LLM)-based triple feature quantification procedure; (2) an objectivity identification module employs pre-trained base models like BERT or RoBERTa that are constrained with the quantified UVO. The experimental result demonstrates our approach can outperform the base models up to 5.91% in objective-F1 and up to 6.97% in accuracy.

## 1. Introduction

The proliferation of online instant communication platforms, such as TikTok, has led to the emergence of large volumes of informal short texts [1]. These texts are often found in social media posts, instant messaging, and online comments, which are characterized by their informal language and brevity [2]. Unlike well-organized formal documents, informal short texts often contain slang [3], abbreviations [4], emoticons [5], non-standard grammar usages [6], and ambiguous language [7], which can hinder accurate information extraction and comprehension [8]. For instance, during the COVID-19 pandemic, tweets like “COVID vaccines alter DNA” spread rapidly as pseudo-factual claims, exploiting informal syntax to evade detection by traditional fact-checking tools [9]. Consequently, it has become a considerable challenge for crowds to identify the objective contents that are the carriers of reliable messages and factual statements from such texts [10,11]. Normally, subjective informal short texts are often laden with personal emotions, opinions, and abbreviations and may exhibit incomplete sentence structures, leading to crowds suffering from identifying the relationships between the relational triplet (a.k.a., subject, predicate, and object) under an uncertain context [12]. However, objective informal short texts, despite being able to be used in informal scenarios, are rooted in factual information and provide accurate, clear communication for knowledge expression and convey. In this case, the goal of differentiating objective informal short texts from subjective ones is quite arduous, especially in the domains where misinformation or inappropriate content can spread rapidly [13].

Objectivity detection in informal texts intersects with but critically diverges from other established NLP tasks such as sentiment analysis [14], stance detection [15], fact verification [16], and opinion mining [17]. For example, sentiment analysis aims to identify the polarity of texts as positive, negative, or neutral; stance detection tries to classify support/opposition toward a target; fact verification tries to validate claims against external evidence; and opinion mining aims to extract subjective expressions from statements. Objectivity detection distinguishes factual claims from non-factual content by evaluating whether statements are grounded in verifiable facts, leveraging syntactic markers (e.g., third-person subjects, definitive tenses) as internal indicators of factuality while addressing the unique challenge of informal texts where opinions and factual phrasing are intricately blended, necessitating deeper structural analysis to disentangle objective assertions from subjective framing. One of the primary challenges in identifying objectivity from informal short texts is their lexical sparsity, where the limited number of words in each text hinders the application of traditional frequency-based or context-dependent models [18,19,20]. These models struggle to capture the subtle linguistic cues that indicate objectivity, particularly in informal short texts [21,22,23,24]. Thus, there is an urgent need to explore more effective tactics for objectivity identification in these texts, thereby fully unlocking their potentiality at automating information processing tasks like fact-checking [25,26,27], information filtering [28,29], and content verification [30,31].

Traditional relational triple-relevant tasks primarily aim to structure information from texts for further analysis and knowledge representation to assist the tasks of information extraction [32], knowledge graph construction [33,34], semantic understanding [35], etc. That means the concept of relational triples maintains a foundational possibility that could support a nuanced approach to understanding content in brief and fragmented statements, especially for informal short texts. Specifically, relational triples help deconstruct these fragmented statements into their core components, which allow for a more nuanced understanding of the underlying objectivity in the statement [36]. Moreover, the latent features of relational triples, such as viewpoints and tenses, are especially valuable as they reveal the essential elements needed to determine whether a statement is objective or subjective [37]. For example, first-person pronouns (e.g., “I believe”, “We think”) typically signal subjective opinions, while impersonal subjects (e.g., “Research shows”, “Statistics indicate”) suggest an objective stance. Furthermore, tense is not merely a grammatical marker; in pragmatics, it functions as a tool to frame information in terms of relevance and reliability. Therefore, effective feature representation for relational triples holds significant potential for identifying objectivity from informal short texts.

To explore the effectiveness of the triple features in identifying objectivity, this paper proposes a novel approach that integrates open information extraction (OpenIE) techniques coupled with LLMs aiming to extract these features from informal short texts. Specifically, we extract and quantify the viewpoint of subjects, the tense of predicates, and the viewpoint of objects (UVO) triple features from these texts to provide a structural foundation that can enhance the analysis and comprehension of the texts. This approach prevents the LLM from unintentionally altering the original text’s meaning, thereby preserving the integrity of the input while improving the model’s performance in identifying objective information. To leverage these features, we embed them into various fine-tunable pre-trained language models, such as RoBERTa and BERT, to verify the effectiveness of the features in detecting objective content within informal short texts.

In summary, this study proposes a novel method aiming at detecting objectivity in informal short texts, which relieves the inherent challenges posed by the informal and sparse nature of these texts. The contribution of this study is summarized as follows.

- This work originally formalizes objectivity detection as a distinct task tailored to the challenges of informal language. Meanwhile, we systematically analyze textual objectivity in platforms like social media and instant messaging.
- We propose a rule-based UVO (subject–predicate–object) quantification framework, which integrates syntactic analysis, tense detection via POS tagging with large-language-model-augmented triple extraction.
- Our two-stage framework synergizes OpenIE, LLMs, and fine-tuned pre-trained encoders. We mitigate hallucination risks inherent in LLMs by decoupling interpretable triple extraction from neural classification via rule-based and LLM voting while preserving input integrity.
- We rigorously evaluate our method across three benchmark datasets and achieve up to 6.97% accuracy improvements over baseline models. The insights revealed in our work provide actionable guidelines for domain-specific adaptation of objectivity detection systems.

## 2. Related Works

### 2.1. Early Objectivity Identification

Objective identification could be treated as a derivative of subjectivity learning known as affective computing or sentiment analysis. Differing from subjective learning, the key to objective identification lies in accurately locating fact-based statements, which is crucial for low false tolerance applications. Early research on subjectivity learning primarily focused on lexical or syntax-based methods. In one of the foundational works by [38] on subjectivity classification, an annotation framework was proposed to classify sentences as subjective or objective with the assistance of multiple annotators. These efforts ensured consistency in text annotation and set a solid groundwork for future research. Building on this, Riloff and Wiebe [39] proposed a more systematic learning framework that automatically extracted subjective expressions from texts, which highlighted the complementary nature of objective identification.

As research advanced, objective identification moved beyond simple lexical features to incorporate broader contextual and grammatical information. Afterward, Wiebe et al. [40] introduced a hybrid approach that combined rule-based and statistical methods. This approach analyzed sentence structure, verb types, and noun specificity to more accurately distinguish objective statements from subjective ones, which provided valuable insights for identifying objectivity.

### 2.2. Objectivity Identification via Artificial Intelligence

With the development of deep learning and machine learning, subjectivity learning evolved into more complex supervised learning models, significantly improving objective identification. For example, Hu and Liu [41] employed a support vector machine (SVM) to classify product reviews as subjective or objective, which effectively vectorizes text features to distinguish opinions from factual descriptions via SVM. Liu et al. [42] used decision trees to build a hierarchical structure for subjective and objective classification, but this method struggled with capturing objective features in complex linguistic patterns. The neural networks, such as CNNs, RNNs, and LSTM also demonstrate excellent performance in objective classification by automatically learning and extracting features from texts. For example, Zhang et al. [43] highlighted the effectiveness of LSTM in capturing logical relationships within long texts, which is beneficial for objective identification in sentences. Additionally, Hu et al. [44] leveraged RNNs to capture contextual information and sequence modeling in text classification, which particularly emphasizes the advantages of RNN in handling sequential data.

Since the rise of Transformer architecture [45], the pre-trained language models, such as BERT [46], RoBERTa [47], T5 [48], BART [49], and especially the series of GPT [50,51,52,53,54], have opened new possibilities for objectivity identification. Qasim et al. [55] demonstrated that fine-tuned BERT models perform exceptionally well in sentiment analysis and other text classification tasks, showing potential for their application in subjectivity classification. These models excel at understanding textual context, which significantly enhances classification accuracy. Larger language models, including GPT-4 [54] and LLaMA [56], have also been applied to text classification tasks. Sun et al. [57] explored the effectiveness of large language models in text classification, showcasing their superiority in capturing context and understanding semantics.

### 2.3. Objectivity in Informal Short Texts

Despite the success of these advanced models, objective identification in short texts presents unique challenges due to informal short texts increasingly incorporating informal and non-standard language [58]. Specifically, informal texts often lack sufficient contextual information and may contain ambiguous information leading to obscuring the traditional subject–object–predicate relationships that underpin many text classification models [59]. This case has prompted researchers to explore new ways to address the challenges posed by the phenomenon of “colloquialism”. Recent advances have leveraged pre-trained language models to handle informal texts, which can contextualize sparse and unstructured text to infer the intent and factuality behind statements [60,61,62]. However, these generative models struggle with short informal texts as they tend to “fill in the gaps” by completing incomplete statements, which may lead to altered meanings and reduced precision in identifying objectivity. Thus, it remains a critical challenge when adopting LLMs in objective identification in short informal texts.

In summary, detecting objectivity in informal short texts remains a challenging task due to their informal nature [63,64] and lexical sparsity. Traditional feature engineering methods [65] have proven inadequate, and while deep learning and pre-trained models have improved performance, they still encounter significant limitations. The ongoing research continues to explore hybrid methods that combine advanced neural networks with syntactic and semantic extraction techniques to better capture the nuances of informal language and improve objectivity detection in short texts [66]. One of the possible solutions aiming at objectivity detection in short informal texts is to investigate the latent information implied in relational triples by combining the nature of objectivity in text. This study is supposed to investigate the effectiveness of the latent features of the relational triples—viewpoints of subjects, tense of predicates, and viewpoints of objects in the task. The details of our methodology design are demonstrated in the following two sections.

## 3. Feature Extraction and Quantification

In this section, we describe the systematic procedure for describing objective statements at the sentence level. According to the event expression concept proposed by Zhang et al. [67], a sentence ($s_{i}$) can be represented by an event set that maintains one or more events (i.e., $E_{i}=\left\{e_{i1},e_{i2},\ldots ,e_{im}\right\}$), where each *event* is a triple set consisting of a subject, a predicate, and an object ($e_{ij}=\left\{u_{ij},v_{ij},o_{ij}\right\},1\leq j\leq m$). Thus, we select subjects (*u*), predicates (*v*), and objects (*o*) of sentences in the form of raw elements as a way to represent sentence-level documents. We subsequently apply the viewpoint of a subject, the tense of a predicate, and the viewpoint of an object as fundamental features to investigate the efficacy of these three parameters in identifying objective cues in short texts. Notably, while our framework processes emojis as text tokens, we acknowledge that visual elements like emojis or images in informal texts may carry additional contextual or tonal cues critical for objectivity determination. Future extensions of this work will explore multimodal integration (e.g., emoji semantic embeddings or image captioning) to address this limitation.

### 3.1. Implementation Overview

**Figure 1:**
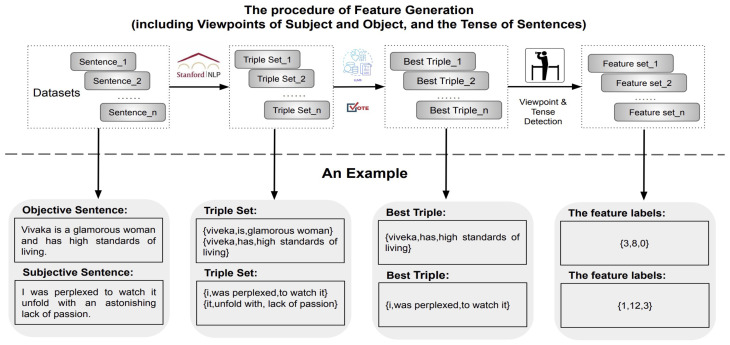
The procedure of UVO feature extraction (including viewpoints of subject and object and the tense of predicate in a short text) is demonstrated using an example.

A complex sentence may include multiple triples, which poses a greater challenge in terms of analysis. Moreover, it is crucial to select the most suitable triple from the triple candidates to represent the sentence for accurate semantic analysis. To this end, we apply a sequence of advanced NLP techniques for the best triple selection and the triple-feature quantification, as shown in Figure 1.

Specifically, we initially extract triples from a sentence using the OpenIE of Stanford NLP. As the example shown in Figure 1, there are two triples—*{vivaka, is, glamorous woman}* and *{vivaka, has, high standards of living}*, that can be obtained from the complex objective sentence—*{’Vivaka is a glamorous woman and has high standards of living’}* using Stanford OpenIE. Next, we leverage six LLMs including source-opened *OLlama 3 70B* (https://ollama.com/library/llama3:70b, accessed on 26 April 2025), *Mistral 7B* (https://huggingface.co/mistralai/Mistral-7B-v0.1, accessed on 26 April 2025), *WizardLM 2 7B* (https://huggingface.co/dreamgen/WizardLM-2-7B, accessed on 26 April 2025), *Phi-3 14B* (https://huggingface.co/microsoft/Phi-3-medium-128k-instruct, accessed on 26 April 2025), *Gemma 2 9B* (https://huggingface.co/google/gemma-2-9b, accessed on 26 April 2025), and *Zhipu AI* (https://www.zhipuai.cn/, accessed on 26 April 2025) to select the most representative triple from the set of OpenIE-extracted candidates through a simple voting mechanism. During this selection, LLMs prioritize triples where the predicate’s tense aligns with temporal cues identified in the sentence (e.g., flagging mismatches like “Back in the day, I go there daily” for reanalysis). The selected triple best captures the main structure and semantics of the original sentence. These models collectively span a diverse range of capabilities, from instruction tuning to general-purpose dialogue, thereby offering multi-perspective judgment for triple selection. The prompt used to select the best triple from the triple sets is shown in Table 1, where the prompt takes the original sentence $s_{i}$ and the generated triple set $E_{i}$ as inputs coupled with a goal and three constraints. Then, we identify the viewpoints of the subject and object and the tense of the predicate embraced in the best triple using our presented rule-based methods. Finally, the viewpoints of the subject and object and the tense of the predicate will be used to facilitate the identification of objective statements.

### 3.2. The Detection of Viewpoints and Tense

To reveal the usability of the viewpoints of the subject and object and the tense of the predicate in discovering objective short informal texts, we design a sequence of rule-based methods to quantify these latent features.

To cover all possible subject- and object-related cases of sentences, we introduce a set, P={0,1,2,3,4}, where first viewpoints, second viewpoints, third viewpoints, and the other cases using non-pronoun named entities (including slang terms like “doggo”) are represented as 1, 2, 3, and 4, respectively. Value 0 in set *P* indicates that no subject or object is identified from input sentences caused by the phenomenon of *“Ellipsis"*. Sentence $s_{i}$ is expressed by the best triple set $e_{i}=\left\{u_{i},v_{i},o_{i}\right\}$, where $u_{i}$ denotes a subject, $v_{i}$ represents a predicate, and $o_{i}$ is an object. Function $F_{view}$ returns the viewpoint of subject $u_{i}$ - $p_{i}^{u}$ and the viewpoint of object $o_{i}$ - $p_{i}^{o}$. Both $p_{i}^{u}$ and $p_{i}^{o}$ must belong to the value set *P*. More formally, we have

(1) $$\begin{array}{c} F_{view}\left(u_{i},o_{i},L_{1},L_{2},L_{3},NER\right)=\left(p_{i}^{u},p_{i}^{o}\right),\left\{p_{i}^{u},p_{i}^{o}\right\}\in P \end{array}$$

where $L_{1}$, $L_{2}$, and $L_{3}$ are manually collected word vocabularies that are comprised of all pronouns in first viewpoints, second viewpoints, and third viewpoints, respectively. NER refers to an existing named entity recognition method. If neither a subject nor an object of sentence $s_{i}$ is initially discovered, then $p_{i}^{u}$ or $p_{i}^{o}$ is tentatively set to 0. When sentence $s_{i}$ embraces a subject (an object), the value of $p_{i}^{u}$ ($p_{i}^{o}$) should be set to 1, 2, 3, or 4.

### 3.3. Tense Detection

To quantify the tense of the predicate in a sentence, we extend three basic tenses—‘future’, ‘present’, and ‘past’—into twelve tenses using three tense-based parameters—‘Perfect’, ‘Continuous’, and ‘Continuous Perfect’. Such twelve tenses cover all the sentence cases summarized in Table 2 accompanied with the POS tagging schemes.

Let Q={1,2,3,4,5,6,7,8,9,10,11,12,0} denote a set of twelve tenses containing future perfect continuous tense (i.e., 1), future perfect tense (i.e., 2), future continuous tense (i.e., 3), future simple tense (i.e., 4), present perfect continuous tense (i.e., 5), present perfect tense (i.e., 6), present continuous tense (i.e., 7), present simple tense (i.e., 8), past perfect continuous tense (i.e., 9), past perfect tense (i.e., 10), past continuous tense (i.e., 11), past simple tense (i.e., 12), and undetected tense (i.e., 0). We devise function $T_{tense}$, which returns tense $q_{i}$ of the best triple (i.e., $e_{i}=\left\{u_{i},v_{i},o_{i}\right\}$). To address temporal cues expressed lexically (e.g., “back in the day”), we supplement the POS-based tense detection with a rule-based filter that maps common temporal adverbs/phrases (e.g., “yesterday”, “currently”) to their implied tenses using a predefined lexicon derived from the TempEval-3 corpus [68]. For instance, the phrase “back in the day” triggers a past-tense override regardless of verb form. Intuitively, the value of $q_{i}$ is an element in set *Q*. More specifically, we have

(2) $$\begin{array}{c} q_{i}=T_{tense}\left(v_{i},schemes\right),q_{i}\in Q. \end{array}$$

where schemes is a rule-based POS tagging detector used to discriminate the tense of the predicate $v_{i}$, as demonstrated in Table 2. More detailed, we employ the *CoreNLP* POS tagger approach [69], which is nested in the Stanford CoreNLP toolkit to identify the part of speech of words. The tense of $q_{i}$ will be returned according to the index of the scheme list if predicate $v_{i}$ has a match within the schemes, To handle non-standard or dialectal verb forms (e.g., “ain’t”), we rely on lexical normalization within the POS tagging phase and apply fallback rules leveraging temporal adverbs (e.g., “yesterday”, “currently”) for tense inference. For slang predicates (e.g., “yeet”), the LLM-aided triple selection (Section 2.1) ensures contextual role inference even if POS tagging mislabels tense; otherwise, $q_{i}$ is set to 0, meaning that no tense is diagnosed in the case.

## 4. System Framework

To verify the effectiveness of the above features extracted from the data processing procedure (the viewpoints of subject and object, the tense of predicate) on objectivity identification, we deploy a local short-text learning system using text encoders aiming at objectivity detection. Specifically, the system framework embraces a text representation module, a triple-feature learning module, and a classification module.

### 4.1. Text Representation

Given a randomly selected sentence $s_{i}$, the true label $y_{i}$, and the corresponding generated triple-features including the viewpoint of subject $p_{i}^{u}$, the tense of predicate $q_{i}$, and the viewpoint of the object $p_{i}^{o}$, we initialize three pre-trained encoders, namely, subject encoder, predicate encoder, and object encoder for the feature representation. More specifically, the subject encoder firstly encodes $s_{i}$ as a sequential representation $F_{i}^{u}=\left\{h_{i1}^{u},h_{i2}^{u},\ldots ,h_{in}^{u}\right\}$ (n is the length of sentence $s_{i}$) and then acquires the global representation of the subject’s viewpoint—$f_{i}^{u}$—via an attention block, same as the predicate encoder and object encoder. Thus, we have

(3) $$\left\{\begin{array}{c} f_{i}^{u}=Attention\left(E_{u}\left(s_{i}\right)\right),\\ f_{i}^{v}=Attention\left(E_{v}\left(s_{i}\right)\right),\\ f_{i}^{o}=Attention\left(E_{o}\left(s_{i}\right)\right), \end{array}\right.$$

where E() is a specific text encoder (subject encoder $E_{u}$, predicate encoder $E_{v}$, or object encoder $E_{o}$) used for obtaining the sequential representations of text—$\left\{F_{i}^{u},F_{i}^{v},F_{i}^{o}\right\}$. It is worth noting that the three Encoders are trainable with the fine-tuning technique by complying with the two restrictions—the triple-features ($p_{i}^{u}$, $q_{i}$, or $p_{i}^{o}$) extracted from the procedure of UVO feature generation and the original true label of text $y_{i}$.

### 4.2. Triple-Feature Learning

**Figure 2:**
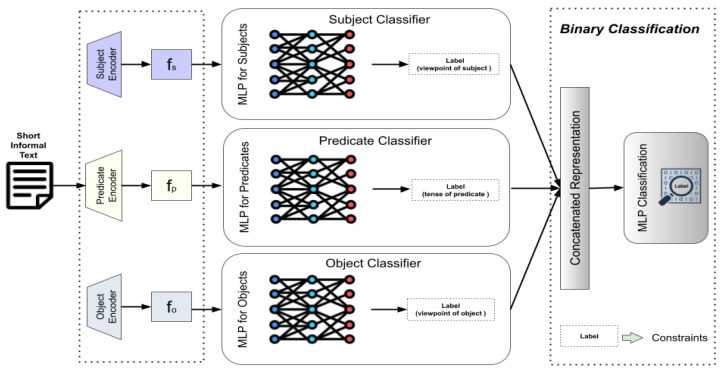
The system framework of objectivity identification. The labels used in three classifiers are generated from the procedure of UVO feature generation (including viewpoints of subject and object and the tense of predicate).

The triple-feature learning module is designed to assist in identifying objective statements. We leverage the three features extracted in the feature generation procedure, including (1) the viewpoint of subject $p^{u}$, (2) the tense of predicate *q*, and (3) the viewpoint of the object $p^{o}$ to train the three local deployed MLPs. As demonstrated in Figure 2, the MLPs are deployed to learn each of the three features. Moreover, the MLPs take the global representations of text as input and then utilize the value of each feature as the true label for parameter training. The loss function for learning the feature (i.e., the viewpoint of the subject $u_{i}$) is presented as follows:

(4) $$L_{u}=\mathrm{CrossEntropy}\left(p^{u},\mathrm{MLP}\left(f^{u}\right)\right)$$

where $f^{u}$ is the global representation calculated by feeding $F^{u}$ into the attention block. It is noteworthy that the value of $p^{u}$ is transformed with one-hot encoding. Moreover, the MLPs’ loss for learning the predicate tense and the object’s viewpoint are denoted as $L_{v}$ and $L_{o}$.

### 4.3. Binary Classification

Once the forward inference processes of the three MLPs are accomplished, the representations of the last hidden layers from the MLPs are concatenated as the final representation of the input sentence. Formally, the final representation of sentence *s* is represented as $f=(f^{u}⊕f^{v}⊕f^{o})$. Finally, we fed *f* into another MLP to approach the true label of the sentence—*y*. Thus, the learning objective of the last MLP is denoted as the following loss function.

(5) $$L_{s}=\mathrm{MSE}\left(y,\mathrm{MLP}\left(f\right)\right)$$

where *f* is the final representation of sentence *s*, and it goes through an MLP to match the dimensionality of the true label for loss calculation. Thus, the total loss of the entire model is as follows.

(6) $$L_{\mathrm{final}}=L_{u}+L_{v}+L_{o}+L_{s}$$

## 5. Experimental Results

In this section, we conduct two kinds of experiments by varying the three extracted features, namely, (1) viewpoints of subjects; (2) tenses of predicates; and (3) viewpoints of objects. The first group experiment aims to evaluate the effectiveness of the three-element variable set ($p^{o}$, *q*, $p^{o}$) in improving the performance of base models. In the second experiment group, we evaluate the impact of each of these three parameters in distinguishing objective sentences by constructing three one-elemental variable sets (i.e., ($p^{u}$),(*q*), and ($p^{o}$)).

### 5.1. Experimental Configurations

**Dataset**: In this study, we adopt three existing datasets to conduct our experiment: (1) the movie review dataset that encompasses 5000 movie review snippets from Rottentomatoes (www.rottentomatoes.com, accessed on 26 April 2025) as a subjective data collection and 5000 objective data items from the plot summaries available from the Internet Movie Database (www.imdb.com); (2) the SST dataset (https://nlp.stanford.edu/sentiment/, accessed on 26 April 2025), also known as Stanford Sentiment Treebank dataset, which are labeled in five fold. Specifically, SST consists of 11,855 single sentences extracted from movie reviews including 1510 very negative, 3140 negative, 2242 neutral, 3111 positive, and 1852 very positive sentences; (3) the Twitter US Airline Review dataset from Kaggle that consists of 2349 sentences labeled in positive, 3073 neutral data points, and 9131 negative data points (https://www.kaggle.com/datasets/crowdflower/twitter-airline-sentiment/data, accessed on 26 April 2025). To adapt the setting of our tasks, we treat data points labeled as ’Neutral’ as proxies for objective data, while acknowledging that this approach may conflate non-polar opinions with factual objectivity [13]. For instance, subjective but neutral statements (e.g., “I think the event was average”) could be mislabeled. To assess label fidelity, we conducted a post hoc analysis on 500 randomly selected samples, finding that 78% of ’neutral’ labels aligned with true objectivity (e.g., factual statements like “The meeting starts at 3 PM”). All of the others were subjective data for the SST and the Airline Review datasets.

**Baselines**: We adopt four pre-trained language models to tier up our proposed method for its effectiveness verification.

- BERT, a bidirectional transformer-based model developed by Google, which is pre-trained using masked language modeling (MLM) and next sentence prediction (NSP) tasks. BERT can read an input sequence from two sides to capture deep contextual information, which allows it to be adaptable for our tasks.
- RoBERTa is developed by Facebook AI, which is an optimized variant of BERT. It removes the next sentence prediction (NSP) task, uses dynamic masking, and is trained on much larger datasets for more epochs than BERT.
- BART is a transformer model also developed by Facebook AI that combines BERT’s bidirectional encoding coupled with GPT’s autoregressive decoding. Its encoder–decoder architecture makes it versatile for both natural language comprehension and generation.
- T5, developed by Google, is a unified framework that can convert NLP tasks into a text-to-text problem. It uses a transformer-based encoder–decoder architecture to handle diverse tasks. Its text-to-text approach simplifies task design and has achieved strong performance across a variety of benchmarks.
- GPT-2, developed by OpenAI, is a large-scale transformer-based language model trained on a massive corpus of internet text in an unsupervised manner. Unlike encoder–decoder frameworks, GPT-2 uses a unidirectional decoder-only architecture, generating text by predicting the next word in a sequence.

**Implementation Details**: In our experiments, we set up fixed hyper-parameters, including the learning rate in Adam optimizer (i.e., $1^{-5\times 10}$), dropout rate before the classification layer (i.e., 0.3), and the number of epochs (i.e., 5 times) across all the datasets. In more detail, each dataset is divided as 80% for training and 20% for testing. We adopt various metrics among all the methods to evaluate the performance including training loss (Tr-Loss), training accuracy (Tr-Acc), testing loss (Te-Loss), testing accuracy (Te-Acc), and the F1 score for objective data (Obj-F1), as shown in Table 3 and Table 4. All the results from the tables are the average score of five epochs. Finally, the objective of this study is to evaluate the effectiveness of the extracted UVO features in identifying objectivity from informal texts by taking advantage of fine-tuning three kinds of pretrained language models.

### 5.2. The First Group Experiment

Table 3 presents the performance comparison between the UVO-added base models and the UVO-free baselines on the three datasets. As shown in the table, UVO-added base models significantly outperform the base models without UVO on every dataset in terms of all the metrics, which demonstrates the effectiveness of triple features in identifying objective sentences. Specifically, we have the following observations from Table 3.

1. Training Efficiency Improvement: In all datasets, the addition of UVO features consistently reduces the training loss as well as yields better training accuracy across all models, which suggests that these features help the models converge better and capture the structure of the data more effectively. In particular, the Bert + UVO model shows the largest reduction in training loss on the Movie dataset, which decreased by 36.69% compared with the base Bert model. Moreover, the Bert + UVO model demonstrated the highest increase in training accuracy percentage increment (6.65%) compared with the corresponding base model on the SST dataset. Notably, even for the GPT2 model—which generally shows higher training loss and lower accuracy compared to other models—the addition of UVO features reduces the training loss significantly across all datasets. For example, in the Movie dataset, the training loss decreased from 0.565 to 0.503, while training accuracy improved from 0.684 to 0.731, indicating better convergence due to the structural cues provided by UVO.
2. Better Generalization on Testing Phase: For all the base pre-trained models, UVO features can help them to achieve both better testing loss and accuracy, which reflects the better generalization capabilities of UVO triple features. This improvement signifies that the UVO-added base models are not overfitting to the training data but learning meaningful patterns that transfer well to the test data. For example, the above-mentioned Bert + UVO model on the Movie dataset also can reduce the testing loss by 1.1% and enhance the testing accuracy by 0.8% under the promise of effective training progress. It is noteworthy that the most significant reduction in testing loss (i.e., 16.18%) is from the UVO-added Bert model on the SST dataset compared with the Bert-only, and the UVO-added Bert model also produces the best testing accuracy improvement (i.e., 4.03%) while tested on the SST dataset. In addition, GPT2 models also exhibit consistent improvements in generalization: on the SST dataset, testing loss decreased from 0.538 to 0.460 and test accuracy increased from 0.784 to 0.811 with UVO. A similar trend is observed on the Twitter dataset, where testing accuracy rose from 0.808 to 0.811 and testing loss slightly reduced, demonstrating that GPT2 also benefits from UVO in learning transferable patterns.
3. Efficient Objectivity Identification: UVO features consistently improve the Obj-F1 score across all datasets and models, demonstrating that the inclusion of the triple features helps all the base models handle objective information better by focusing on syntactic roles (viewpoints of subjects, tenses of predicates, and viewpoints of objects). For instance, the T5 + UVO model achieves an Obj-F1 of 0.956 on the Movie dataset and 0.926 on the Twitter dataset. Similarly, GPT2 + UVO shows marked improvement over the baseline GPT2 in Obj-F1 scores: from 0.727 to 0.735 (+0.8%) on Movie, from 0.873 to 0.896 (+2.3%) on SST, and from 0.880 to 0.894 (+1.4%) on Twitter, reinforcing the conclusion that UVO helps even relatively weaker baselines like GPT2 to better capture objectivity.

### 5.3. The Ablation Study

To further investigate the impact of each feature in the UVO triple, we conduct two ablation studies by either utilizing or disabling each of the UVO features. We first activate the effect of a single feature on the performance of base models. As shown in Table 4, we verify the efficiency of each of the UVO features on all base pre-trained models across the three datasets. Furthermore, we have the following observations from the table.

1. In the Movie dataset, the viewpoint of subjects (i.e., U) can supply better improvement to the performance in terms of five metrics across all of the base pre-trained models compared with the other two features—the tense of predicates and the viewpoint of objects (i.e., Bert + U achieves the best performance—0.095 training loss, 0.97 training accuracy, 0.102 testing loss, 0.968 testing accuracy, and 0.966 F1 score in objective). Such a phenomenon reflects that the viewpoint of object (O) and the tense of predicate (V) might be less influential than the subject’s perspective in movie reviews. While the viewpoint of objects and the tense of movie reviews are important, they are secondary to how the reviewer personally feels and expresses those feelings. Since the subject’s expression of opinion is critical in movie reviews, its viewpoint allows the model to focus more on this subjective lens, which is key for improving generalization in objectivity detection.
2. It is observable from the results yielded from the SST dataset that the tense of predicates (i.e., V) promotes the base models with the relatively best performance in perspectives of all five metrics compared with the viewpoints of subjects and objects. That means, in the SST dataset, the tense of predicates is a major clue in determining whether the content is a personal opinion or a more neutral statement of fact. More insightful, since SST data consists of shorter texts, and other contextual information is sparse, the verbs carry a significant proportion of the determination in helping the model to identify objectivity.
3. For the Twitter dataset, various pre-trained base models yield relatively unstable performance compared with the experiment results on the other two datasets. However, the viewpoint of subjects (U) still plays the most important role in distinguishing objective texts. Specifically, the different performance of fine-tuning pre-trained models on the Twitter dataset when applying the U, V, or O constraints reflects their architectural differences and varying pretraining objectives. Models like RoBERTa excel in short-text, informal settings like Twitter due to their fine-tuned contextual embeddings, yet BART performs better at tasks involving sequence structure (e.g., tense or object viewpoint detection). It is crucial to understand the differences between models or datasets to detect objectivity in short informal text.

Similar to the first group of ablation study, Table 5 demonstrates the efficiency of each tuple of the UVO features (implemented by disabling each of the UVO features), which reveals that the performance of base models may be varied while each of the UVO features is disabled in different datasets. The analysis for the second ablation study is enumerated as follows.

- In the Movie dataset, combinations of two features—especially U+V and U+O—tend to slightly improve performance compared to using a single constraint. For example, BERT with U+O achieves 0.098 Tr-Loss, 0.969 Tr-Acc, 0.118 Te-Loss, 0.964 Te-Acc, and 0.966 Obj-F1, nearly matching the best single-feature result (BERT+U). This suggests that while subject viewpoint (U) is dominant, integrating it with either tense (V) or object viewpoint (O) can reinforce semantic grounding. However, the marginal improvement indicates that most of the predictive strength still lies in the subject’s viewpoint, highlighting the expressive nature of movie reviews.
- For SST, the dual-feature combinations V+O and U+V perform slightly better than single features for some models. For instance, RoBERTa with V+O yields a Te-Acc of 0.824 and Obj-F1 of 0.903, higher than U or O alone. This reinforces the earlier observation that verb tense (V) carries the most semantic weight in the SST dataset, and when enhanced with object viewpoint (O), the contextual understanding is deepened. Since SST texts are short and less informative, having both predicate tense and object perspectives can aid in identifying subtle subjectivity markers.>
- In Twitter, U+V and U+O combinations generally outperform other pairs or single features. For example, T5 with U+O achieves 0.821 Tr-Acc and 0.915 Obj-F1, higher than V+O. These results imply that in short and informal texts like tweets, integrating subject viewpoint (U) with an additional semantic constraint (V or O) enhances model robustness. However, due to noise and structural variation in tweets, improvements remain dataset- and model-dependent.

## 6. Conclusions

As key elements in sentence structure, viewpoints of subjects, tenses of predicates, and viewpoints of objects can directly influence the tone and objectivity of the text. Inspired by this, we propose to investigate the impact of the three features on objective detection in short informal text. To achieve this goal, we propose an LLM- and OpenIE-based triple feature extraction procedure to quantify each of the features. Upon that, we construct a pre-trained encoder-based model aiming at objectivity detection by leveraging the extracted triple features as the constraints of model training. Our experimental results demonstrate the superiority of our method in identifying objectivity in datasets that consist of short informal text. Moreover, we conduct the corresponding ablation studies to further explore the impact of each of the three triple features on the task, which reflects that each UVO feature has different influences while varying base models or datasets. Although the proposed UVO quantification framework demonstrates strong performance in detecting objectivity in informal texts, the dependence on OpenIE and rule-based filtering may lead to unstable outputs when processing noisy or structurally complex sentences. This can affect the quality of downstream triple selection. Furthermore, our experiments are conducted primarily on social media and sentiment datasets. The framework’s generalizability to highly specialized domains has not yet been evaluated. In the future, our approach will be applied to further investigate the difference in objectivity between human-written text and LLM-generated text.

## Figures and Tables

**Table 1:** The prompt used in LLMs for best triple selection.

text:{text}
triples:{triples}
Goal: Choose the best subject-predicate-object triples from the text.
Requirements 1: The output format should be: (subject, predicate, object).
Requirements 2: Do not rephrase the triples.
Requirements 3: Avoid excessive introduction or background description.

**Table 2:** All twelve tenses accompanied with corresponding POS tagging schemes and labels.

Tenses	POS Schemes	Example	Labels
Future Perfect Continuous	“MD” + “VB” + “VBN” + “VBG”	I will have been doing	1
Future Perfect	“MD” + “VB” + “VBN”	I will have done	2
Future Continuous	“MD” + “VB” + “VBG”	I will be doing	3
Future Simple	“MD”	I will do	4
Present Perfect Continuous	“VBD” + “VBN” + “VBG”	I have been doing	5
Present Perfect	“VBD” + “VBN” + “VBG”	I have done	6
Present Continuous	“VBD” + “VBN”	I am doing	7
Present Simple	“VBD”	I do	8
Past Perfect Continuous	“VBP”/“VBZ” + “VBN” + “VBG”	I had been doing	9
Past Perfect	“VBP”/“VBZ” + “VBN”	I had done	10
Past Continuous	“VBP”/“VBZ” + “VBG”	I was doing	11
Past Simple	“VBP”/“VBZ”	I did	12

**Table 3:** The performance comparison between encoders-only baselines and the triple-feature-based encoders across the three datasets. The triple features, UVO, represent viewpoints of subjects, tenses of predicates, and viewpoints of objects, respectively. The best values in various metrics have been bolded.

Datasets	Methods	Tr-Loss	Tr-Acc	Te-Loss	Te-Acc	Obj-F1
Movie	RoBERTa	0.136	0.953	0.139	0.955	0.954
	RoBERTa + UVO	**0.101**	**0.966**	**0.129**	**0.963**	**0.962**
	Bert	0.101	0.972	0.130	0.958	0.954
	Bert + UVO	**0.086**	**0.975**	**0.117**	**0.965**	**0.963**
	Bart	0.181	0.933	0.133	0.956	0.954
	Bart + UVO	**0.167**	**0.934**	**0.121**	**0.961**	**0.961**
	T5	0.181	0.940	0.122	0.960	0.956
	T5 + UVO	**0.152**	**0.946**	**0.119**	**0.960**	**0.956**
	GPT2	0.565	0.684	0.566	0.702	0.727
	GPT2 + UVO	**0.503**	**0.731**	**0.565**	**0.705**	**0.735**
SST	RoBERTa	0.536	0.765	0.518	0.803	0.889
	RoBERTa + UVO	**0.498**	**0.809**	**0.486**	**0.812**	**0.898**
	Bert	0.520	0.769	0.554	0.786	0.879
	Bert + UVO	**0.450**	**0.816**	**0.464**	**0.817**	**0.898**
	Bart	0.528	0.792	0.554	0.801	0.890
	Bart + UVO	**0.511**	**0.792**	**0.491**	**0.815**	**0.897**
	T5	0.575	0.707	0.551	0.739	0.834
	T5 + UVO	**0.461**	**0.809**	**0.499**	**0.809**	**0.893**
	GPT2	0.497	0.808	0.538	0.784	0.879
	GPT2 + UVO	**0.478**	**0.820**	**0.493**	**0.811**	**0.896**
Twitter	RoBERTa	0.344	0.874	0.353	0.871	0.921
	RoBERTa + UVO	**0.282**	**0.887**	**0.318**	**0.872**	**0.922**
	Bert	0.328	0.875	0.395	0.867	0.919
	Bert + UVO	**0.252**	**0.902**	**0.343**	0.866	**0.919**
	Bart	0.436	0.801	0.401	0.848	0.905
	Bart + UVO	**0.359**	**0.840**	**0.334**	**0.863**	**0.916**
	T5	0.372	0.850	0.335	0.864	0.920
	T5 + UVO	**0.329**	**0.860**	**0.302**	**0.874**	**0.926**
	GPT2	0.471	0.806	0.492	0.808	0.880
	GPT2 + UVO	**0.440**	**0.817**	**0.460**	**0.811**	**0.894**

**Table 4:** The ablation study we conducted for investigating the role of each of the UVO features in affecting the performance of five pre-trained base models on objectivity detection across the three datasets. The best values in various metrics have been bolded.

Datasets	Models	Constraints			Tr-Loss	Tr-Acc	Te-Loss	Te-Acc	Obj-F1
		U	V	O					
**Movie**	**RoBERTa**	✓			**0.131**	**0.959**	0.127	**0.963**	**0.961**
			✓		0.136	0.957	**0.118**	0.961	0.961
				✓	0.142	0.951	0.130	0.958	0.957
	**Bert**	✓			**0.095**	**0.970**	**0.102**	**0.968**	**0.966**
			✓		0.108	0.969	0.139	0.953	0.952
				✓	0.104	0.970	0.112	0.963	0.962
	**BART**	✓			**0.190**	0.932	0.156	**0.949**	**0.948**
			✓		0.220	0.920	0.181	0.939	0.939
				✓	0.194	**0.934**	**0.153**	0.947	0.946
	**T5**	✓			**0.174**	**0.946**	0.135	**0.958**	**0.954**
			✓		0.213	0.926	0.148	0.949	0.944
				✓	0.184	0.939	**0.130**	0.955	0.951
	**GPT2**	✓			**0.502**	0.731	**0.585**	**0.687**	**0.735**
			✓		0.505	**0.733**	0.619	0.676	0.719
				✓	0.504	0.729	0.601	0.684	0.727
**SST**	**RoBERTa**	✓			0.548	0.765	0.524	0.808	0.893
			✓		**0.523**	**0.796**	**0.490**	**0.822**	**0.902**
				✓	0.539	0.773	0.519	0.805	0.890
	**Bert**	✓			0.557	0.764	0.534	0.800	0.890
			✓		**0.528**	**0.781**	**0.514**	**0.806**	**0.891**
				✓	0.543	0.767	0.528	0.789	0.881
	**BART**	✓			0.537	0.774	0.545	0.814	0.897
			✓		**0.515**	**0.797**	**0.494**	**0.827**	**0.905**
				✓	0.518	0.796	0.529	0.813	0.897
	**T5**	✓			0.665	0.603	0.584	0.693	0.780
			✓		**0.521**	**0.766**	**0.506**	**0.800**	**0.887**
				✓	0.526	0.760	0.516	0.798	0.886
	**GPT2**	✓			0.498	0.806	0.507	0.802	0.891
			✓		**0.486**	**0.814**	**0.480**	**0.816**	**0.899**
				✓	0.492	0.811	0.487	0.803	0.891
**Twitter**	**RoBERTa**	✓			**0.317**	**0.877**	**0.321**	**0.873**	**0.923**
			✓		0.349	0.859	0.323	0.868	0.921
				✓	0.360	0.858	0.350	0.863	0.917
	**Bert**	✓			**0.304**	**0.889**	**0.346**	**0.868**	**0.919**
			✓		0.331	0.877	0.347	0.860	0.915
				✓	0.360	0.860	0.364	0.858	0.914
	**BART**	✓			0.409	0.806	**0.356**	**0.856**	0.910
			✓		**0.399**	**0.820**	0.362	0.855	**0.913**
				✓	0.450	0.779	0.395	0.834	0.896
	**T5**	✓			**0.393**	**0.836**	0.350	0.853	0.913
			✓		0.414	0.815	0.366	0.852	0.909
				✓	0.404	0.826	**0.339**	**0.862**	**0.917**
	**GPT2**	✓			**0.440**	**0.819**	0.455	**0.823**	**0.902**
			✓		0.446	0.810	**0.453**	0.816	0.897
				✓	0.462	0.814	0.466	0.808	0.891

**Table 5:** The ablation study we conducted for investigating the role of two of the UVO features in affecting the performance of five pre-trained base models on objectivity detection across the three datasets. The best values in various metrics have been bolded.

Datasets	Models	Constraints			Tr-Loss	Tr-Acc	Te-Loss	Te-Acc	Obj-F1
		U	V	O					
**Movie**	**RoBERTa**	✓	✓		**0.125**	**0.957**	**0.122**	**0.963**	**0.964**
		✓		✓	0.138	0.949	0.133	0.954	0.957
			✓	✓	0.152	0.943	0.135	0.953	0.956
	**Bert**	✓	✓		0.099	0.970	**0.097**	**0.969**	**0.970**
		✓		✓	**0.096**	**0.972**	0.116	0.961	0.964
			✓	✓	0.098	0.969	0.118	0.964	0.966
	**BART**	✓	✓		**0.169**	**0.935**	0.135	0.955	0.955
		✓		✓	0.179	0.930	**0.123**	**0.956**	**0.959**
			✓	✓	0.173	0.934	0.138	0.951	0.952
	**T5**	✓	✓		0.159	0.945	0.119	**0.964**	**0.962**
		✓		✓	**0.157**	0.945	**0.114**	0.962	0.959
			✓	✓	0.160	**0.946**	0.121	0.960	0.957
	**GPT2**	✓	✓		**0.505**	**0.729**	**0.583**	**0.688**	**0.670**
		✓		✓	0.507	0.727	0.615	0.674	0.627
			✓	✓	0.505	0.722	0.598	0.673	0.652
**SST**	**RoBERTa**	✓	✓		0.503	**0.806**	0.478	0.820	0.900
		✓		✓	0.518	0.793	0.497	0.810	0.896
			✓	✓	**0.496**	0.803	**0.475**	**0.824**	**0.903**
	**Bert**	✓	✓		0.497	0.795	0.504	0.802	0.889
		✓		✓	0.488	0.789	0.525	0.798	0.886
			✓	✓	**0.455**	**0.807**	**0.470**	**0.811**	**0.894**
	**BART**	✓	✓		0.522	0.780	**0.511**	**0.818**	**0.900**
		✓		✓	0.526	0.774	0.539	0.803	0.889
			✓	✓	**0.508**	**0.798**	0.515	0.802	0.889
	**T5**	✓	✓		0.471	0.807	**0.482**	0.812	0.895
		✓		✓	0.466	0.810	0.491	0.812	0.895
			✓	✓	**0.461**	**0.811**	0.483	**0.813**	**0.896**
	**GPT2**	✓	✓		0.493	**0.811**	0.496	0.805	0.892
		✓		✓	**0.493**	0.810	0.496	0.804	0.892
			✓	✓	0.494	0.809	0.482	0.815	0.897
**Twitter**	**RoBERTa**	✓	✓		**0.296**	**0.883**	**0.302**	**0.878**	**0.927**
		✓		✓	0.304	0.878	0.315	0.877	0.925
			✓	✓	0.335	0.857	0.344	0.861	0.915
	**Bert**	✓	✓		**0.267**	**0.894**	0.336	0.869	0.920
		✓		✓	0.297	0.880	0.355	0.858	0.913
			✓	✓	0.294	0.879	**0.323**	**0.876**	**0.925**
	**BART**	✓	✓		0.364	0.844	**0.309**	**0.878**	**0.927**
		✓		✓	0.397	0.813	0.329	0.863	0.916
			✓	✓	**0.360**	**0.847**	0.309	0.874	0.925
	**T5**	✓	✓		0.359	0.846	0.318	0.869	0.923
		✓		✓	0.385	0.821	0.335	0.862	0.918
			✓	✓	**0.334**	**0.862**	**0.314**	**0.871**	**0.925**
	**GPT2**	✓	✓		0.465	0.808	0.453	**0.819**	**0.899**
		✓		✓	**0.463**	**0.809**	0.461	0.813	0.895
			✓	✓	0.468	0.807	**0.451**	0.814	0.895

## Data Availability

The data presented in this study are available on request from the corresponding author.
